# The Time-Lag Effect of Climate Factors on the Forest Enhanced Vegetation Index for Subtropical Humid Areas in China

**DOI:** 10.3390/ijerph20010799

**Published:** 2023-01-01

**Authors:** Jushuang Qin, Menglu Ma, Jiabin Shi, Shurui Ma, Baoguo Wu, Xiaohui Su

**Affiliations:** 1 School of Information Science and Technology, Beijing Forestry University, Beijing 100083, China; 2 Engineering Research Center for Forestry-Oriented Intelligent Information Processing, National Forestry and Grassland Administration, Beijing 100083, China; 3 Research Institute of Forestry Informatization, Beijing Forestry University, Beijing 100083, China

**Keywords:** TVP-VAR model, time-varying impulse response, time-lag effect, enhanced vegetation index, climate factors, forest ecological station

## Abstract

Forests represent the greatest carbon reservoir in terrestrial ecosystems. Climate change drives the changes in forest vegetation growth, which in turn influences carbon sequestration capability. Exploring the dynamic response of forest vegetation to climate change is thus one of the most important scientific questions to be addressed in the precise monitoring of forest resources. This paper explores the relationship between climate factors and vegetation growth in typical forest ecosystems in China from 2007 to 2019 based on long-term meteorological monitoring data from six forest field stations in different subtropical ecological zones in China. The time-varying parameter vector autoregressive model (TVP-VAR) was used to analyze the temporal and spatial differences of the time-lag effects of climate factors, and the impact of climate change on vegetation was predicted. The enhanced vegetation index (EVI) was used to measure vegetation growth. Monthly meteorological observations and solar radiation data, including precipitation, air temperature, relative humidity, and photosynthetic effective radiation, were provided by the resource sharing service platform of the national ecological research data center. It was revealed that the time-lag effect of climate factors on the EVI vanished after a half year, and the lag accumulation tended to be steady over time. The TVP-VAR model was found to be more suitable than the vector autoregressive model (VAR). The predicted EVI values using the TVP-VAR model were close to the true values with the root mean squares error (RMSE) < 0.05. On average, each site improved its prediction accuracy by 14.81%. Therefore, the TVP-VAR model can be used to analyze the relationship of climate factors and forest EVI as well as the time-lag effect of climate factors on vegetation growth in subtropical China. The results can be used to improve the predictability of the EVI for forests and to encourage the development of intensive forest management.

## 1. Introduction

In recent years, the greenhouse effect has intensified, and the frequency of extreme weather has increased [1,2,3]. In the context of global climate change, the analysis of vegetation dynamics and responses has become a hot research topic [4,5]. Forests respond to climate change with high sensitivity [6]. Forest structure [7,8,9], vegetation cover [10], and vegetation phenological period [11,12] are closely related to meteorological factors such as temperature, precipitation, and solar radiation. In 1999, China established forest ecological observation stations using the most representative forest ecosystem types and regions. The research at these sites focuses on global climate change and sustainable forestry development and aims to reveal the relationship between the structure and function of the forest ecosystem and its modes of dynamic change. These sites are also an effective way to study the forest ecosystem’s critical processes and to seek strategies for sustainable and rational management of forest resources [13,14]. There are differences in the characteristics of climate change and differences in the way vegetation responds to the climate in various ecological zones. Therefore, it is essential to conduct vegetation climate response studies at field stations in different ecological zones in order to best manage forests. Forest managers can formulate scientific and reasonable policies by monitoring and evaluating the dynamic changes in forest vegetation. The ultimate goal of this work would be to improve the adaptability of the forest ecosystem to climate change and to improve the resilience of forests to disasters. At the same time, this work provides a basis for accurate measurement and monitoring of forests as an effective carbon sink.

The vegetation index is one of the significant remote sensing parameters reflecting vegetation growth status. The commonly used remote sensing vegetation indices include the Normalized Difference Vegetation Index (NDVI) and the Enhanced Vegetation Index (EVI). The NDVI can well reflect vegetation growth and ground vegetation coverage. However, it is prone to saturation in areas with high vegetation coverage, and its ability to resist atmospheric noise is weak. The EVI can reduce the influence of both atmospheric and soil noise, overcome the shortcomings of NDVI, and provide a sound foundation for quantitative remote sensing research in densely forested areas [15]. Using EVI data from an experimental area in Guangdong from 2001 to 2018, Wang and Fan [16] analyzed the spatial and temporal patterns of vegetation activity in the experimental area. They established a perturbation lag model between the EVI and climate factors. Udelhoven et al. [17] used a distributed lag model to assess the effect of 16 consecutive days of abnormal rainfall on the EVI in the Okavango watershed and distinguished sensitive and non-sensitive areas of the basin using an applied logistic regression model.

The association between climate conditions and the growth dynamics of vegetation at various time and space scales has been analyzed using a number of techniques by researchers from all over the world. Jin et al. [18] analyzed the correlation between the NDVI and temperature and precipitation in August in Xi’an from 2004 to 2013. They found that precipitation changes have a time-lag effect on vegetation in Xi’an. Ding et al. [19] used multiple linear regression (MLR) methods to explore the time-lag and cumulative effects of temperature and precipitation on vegetation growth and their combined effects on a global scale. Neural network models are also widely used in the study of vegetation growth changes. Lou et al. [20] analyzed the contributions of surface temperature, precipitation, and soil moisture to the dynamic spatial and temporal variations of vegetation at different elevations in the Qaidam Basin using an artificial neural network (ANN) model. Reddy and Prasad [21] proposed a method for forecasting changes in vegetation based on NDVI data and the long short-term memory neural network technique (LSTM). The above studies show that climate has a significant impact on vegetation growth. In the future, this impact will increase with the intensification of climate change. Linear regression methods and neural network models tend to focus only on the numerical values themselves. It is difficult to intuitively explain the intrinsic relationship between vegetation growth and climate change.

A vector autoregressive model (VAR) is an unstructured model. It uses each endogenous variable in the system as a function of the lagged values of all endogenous variables in the system to construct a model [22]. Redlin and Gries [23] used this model to study the impact of various components of the global carbon budget on climate change. Bruns et al. [24] used this model to simulate the synergistic role of the ocean in global climate change. Although some studies have used the VAR model to solve ecological problems, the model is essentially a constant parameter model, and it is difficult to describe the time-varying characteristics driven by endogenous variables. When the events that occur are not within the historical data that the VAR model relies on, the accuracy of the model will be greatly biased [25,26]. Unlike the VAR model with fixed parameters, the TVP-VAR model can simulate the impulse response of different lag periods over time [27]. In summary, the existing research results provide sufficient theoretical basis for the basic hypothesis of this study. Changes in the EVI are mutually influenced by climate variables such as temperature and light, while climate variables are primarily governed by the seasons. Existing research on the time-delay effect of climate factors is primarily limited to global static analyses. The interaction between variables and the evolution of a process over time cannot be reflected by simple moving average methods or linear models [28,29]. Therefore, the study chooses the TVP-VAR model, which has the flexibility to analyze the dynamic response characteristics of the EVI at different lead times and time points. This model is also able to decompose the overall contribution of various factors that promote vegetation growth into several different mechanisms, that is, accurately estimate the duration and intensity of the time-lag effects of different meteorological factors.

The goals of this study were: (1) to quantify the time delay effect of climate change on vegetation growth though establishing the TVP-VAR model; (2) to reveal the convergence and divergence of vegetation growth in different growth seasons in field stations of typical subtropical forest ecosystems through the empirical observation and quantitative analysis of vegetation response to climate change; and (3) to predict the response of vegetation change based on the VAR and TVP-VAR models, and compare the prediction results to evaluate accuracy.

## 2. Materials and Methods

### 2.1. The Study Area

**Figure 1:**
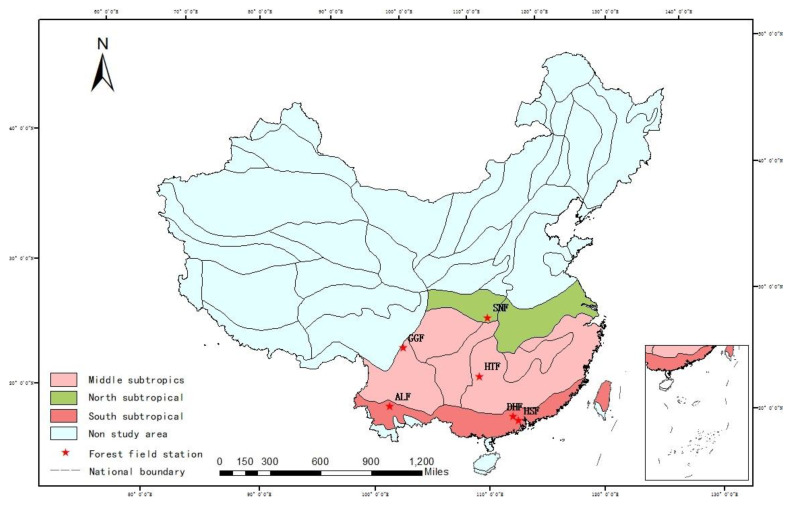
Spatial distribution of forest field stations in humid subtropical regions.

The national field stations were established in 1999, include 8 types, such as forest, grassland, farmland, desert, swamp, lake, and bay ecosystems. There are 17 national scientific observation and research stations for forest ecosystems [30]. According to the 11 temperature zones identified by Yang et al. [31], the 6 stations in this study are distributed in the humid regions of the northern subtropics, middle subtropics, and south subtropics (Figure 1). Basic information about the observation stations is provided in Table 1.

### 2.2. Data Source

#### 2.2.1. Meteorological Data

The forest ecosystem field stations long-term meteorological monitoring data were acquired from the National Ecological Science Data Center Resource Sharing Service Platform (http://www.cnern.org.cn/, accessed on 12 April 2021). The monitoring data were collected by the ground standard meteorological observation field for each forest field station. According to the specifications for construction of long-term observation research stations for the forest ecosystem (GB/T 40053–2021) in China, meteorological observation sites are all set up in flat and open areas in the forest area around the key distribution areas of the forest resources. The setup of all stations must ensure that the observation instruments will not be disturbed by the shadows of surrounding obstacles. Collected meteorological data include meteorological observations and monthly readings of solar radiation, precipitation (PRE; mm), air temperature (TEM; °C), humidity (RHU; %), and photosynthetically active radiation (PAR; mol/m^2^). The time range for the northern subtropical humid region field station (SNF) data is 11 years; for the other stations’ data, the time range is 13 years (Table 2).

#### 2.2.2. Enhanced Vegetation Index (EVI) Data

EVI data were obtained from MOD13Q1 (Version 6), a resolution imaging spectrometer product. Moreover, the data set was acquired in the same period as the field station ecosystem meteorological monitoring data, 2007–2019, with a spatial resolution of 250 m and a temporal resolution of 16 d. The data were downloaded from NASA’s website (https://earthdata.nasa.gov/, accessed on 12 April 2021), and then we used the MRT (MODIS Reprojection Tools, National Center for Supercomputing Applications at the University of Illinois at Urbana Champaign, Champaign-Urbana, USA) for projection conversion, cutting and splicing, and other pre-processing. The Maximum Value Composite (MVC) method was used to obtain monthly EVI data.

#### 2.2.3. Data Set Partition

The time scale of data collection at the field station is in months. When exploring the corresponding differences in vegetation growth in different periods of the growing season, this study arranged the time series data by month and recorded it as Data set 1. This data set was used to longitudinally compare the differences in the lag effect of climate factors in different periods in the same region. In September 2018, China’s coastal areas were hit by Typhoon Mangkhut. The weather caused by the typhoon severely damaged the vegetation in the Dinghushan area [32]. In order to verify the ability of the model constructed in this paper to capture emergencies, when studying the cumulative time lag of climate factors on the EVI, this paper took 2018 as a time node on the basis of monthly time series data and divided the time span into two periods. The data from 2007 to 2018 were recorded as Data set 2, and the data after 2018 were recorded as Data set 3. Data set 2 was input into the model and impulse response analysis was performed to compare the time lag cumulative differences of climate factors in different regions in the same period. Data set 3 was used as a predictive test set to verify the applicability of the model.

### 2.3. Data Processing and Analysis

**Figure 2:**
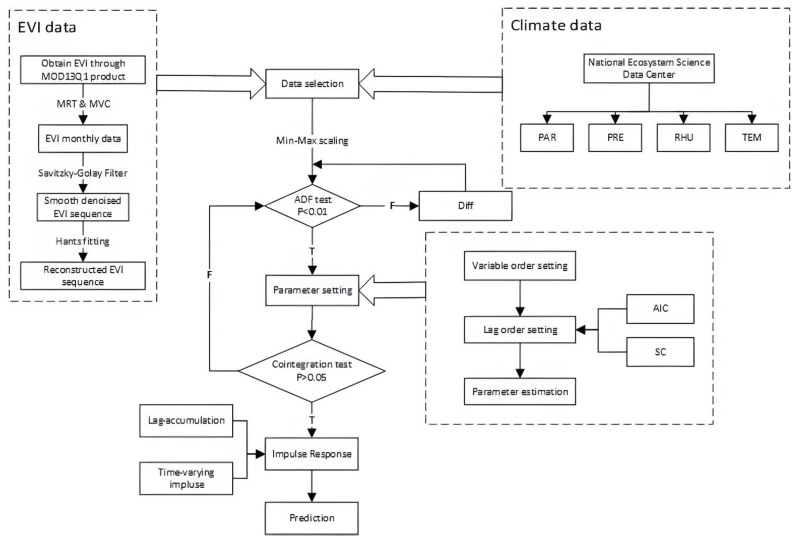
The modeling process of TVP-VAR.

Based on EVI data and meteorological monitoring data, this paper normalized the collected time series data. For data that passed the Augmented Dickey-Fuller (ADF) test, we first determined the order in which the variables enter the model. The order of the model was then determined using the Akaike information criterion (AIC) and Schwartz criterion (SC). According to the above parameters, the TVP-VAR model was initially established. Considering the actual needs of data processing, we used the Markov Chain Monte Carlo (MCMC) method and constructed a Markov chain containing 2000 iterations for the Bayesian estimation. After the optimal parameter combination was obtained, the time-varying impulse response analysis of the model was carried out. Finally, the applicability of the model to forestry was evaluated through the prediction accuracy of the model. The technical process is shown in Figure 2.

#### 2.3.1. Data Pre-Processing

The subtropical region in China is continuously affected by clouds and rain. Unfortunately, the EVI data synthesized by the maximum values can only eliminate the low value noise. Therefore, it was difficult to recover the actual surface situation for this region. However, this was addressed by offsetting the date corresponding to the maximum value [33,34]. The SG-HANTS algorithm was used to smooth the EVI time series data in this study.

The SG filtering method (apart from HANTS) is a weighted average algorithm where the weighted factor depends on the number of least-squares fits to a given higher-order polynomial within the filter window [35] and the expression is as follows:

(1) $$Y_{j}=\overset{i=m}{\underset{i=-m}{\sum }}\frac{C_{i}Y_{j+i}}{N}$$

where $Y_{j+i}$ is the original EVI data; $Y_{j}$ is the value of the EVI time series fitted by the SG filter; $C_{i}$ is the filtering coefficient of the ith time series data; N is the data contained in the sliding window; and m is the size of the filtering window controlling the smoothing effect. The SG filtering method can remove most of the intense noise in the sequence based on preserving the original curve information and is widely used in smoothing and de-noising time-series data [36]. Therefore, harmonic analysis of time series (HANTS) was introduced to reconstruct the original time series [37].

HANTS uses the Fourier transform as the theoretical basis and adds the least-squares method to fit the reconstruction of the original time series with the expression:

(2) $$y_{i}=A_{0}+\overset{m}{\underset{j=1}{\sum }}A_{j}\sin({\bar{w}}_{j}i+\theta_{j}),\;i=0,1,\ldots n$$

(3) $${\bar{w}}_{j}=2j\pi /n$$

where $\;A_{0}$ is the residual term of the harmonic (i.e., the mean value of the series), $A_{j}$ is the amplitude of the harmonic, $\theta_{j}$ is the initial phase of the harmonic, n is the length of the sequence, ${\bar{w}}_{j}$ is the frequency of the harmonic, and m=n−1 is the number of harmonics.

With repeated experiments, the optimal parameters of the HANTS algorithm are determined as follows: the number of frequencies is 2, the fitting error (FET) is 500, and the number of remaining points (DOD) is 8.

In this paper, the original EVI data were processed using the extended tool MRT in ENVI and the MVC method. After that, the EVI monthly value sequence was smoothed out and made less noisy with the SG filtering method. The de-noised EVI time-series data is input into HANTS software to obtain the reconstructed EVI time-series data.

The dimensions of each factor involved in the modeling are different. In order to reduce the influence of different dimensions between data, data normalization processing is required. In this paper, the linear function normalization (Min-Max scaling) method was used to convert the original data to the range of [0, 1]. The specific calculation process is shown in Formula (4), where X and $X^{\prime }$ represent the original-feature data and the normalized-feature data, respectively. $X_{max}$ and $X_{min}$ represent the maximum and minimum values in the original-feature data, respectively. Raw-feature data include EVI, TEM, RHU, PRE, and PAR data sets.

(4) $${X^{\prime }}_{\left(EVI,TEM,RHU,PRE,PAR\right)}=\frac{X-X_{min}}{X_{max}-X_{min}}$$

#### 2.3.2. Bayesian TVP-VAR Model

##### TVP-VAR Model Setting

The TVP-VAR model was first applied in the analysis of monetary policy [38], and the parameters in this model are set to vary over time. Changes in the EVI are affected by multiple climate factors, and climate factors are mostly regulated by seasons. The TVP-VAR model has the advantage of decomposing the overall contribution of the various factors into several different mechanisms. In this paper, taking full advantage of the model, based on the algorithm of Nakajima et al. [27], a five-variable TVP-VAR model consisting of TEM, PRE, RHU, PAR, and EVI was constructed to reveal the influence of climate factors on vegetation growth.

The measuring equation for the TVP-VAR model can be expressed as follows:

(5) $$y_{t}=B_{1}y_{t-1}+\cdots +B_{p}y_{t-p}+A^{-1}\Sigma \varepsilon_{t}$$

where the $y_{t}=\left(EVI_{t},TEM_{t},RHU_{t},PRE_{t},PAR_{t}\right)$, $B_{p}$ represents a time-varying coefficient vector matrix, and $A^{-1}\Sigma \varepsilon_{t}$ is a random error term. In addition to the measuring equation, the TVP-VAR model also includes the spatial state equation used to determine the time-varying parameters. The formulas are expressed as follows:

(6) $$\{\begin{array}{c} \beta_{t+1}=\beta_{t}+\mu_{\beta t}\\ a_{t+1}=a_{t}+\mu_{\beta t}\\ h_{t+1}=h_{t}+\mu_{ht} \end{array}$$

(7) $$\left[\begin{array}{c} \begin{array}{c} \varepsilon_{t}\\ \mu_{\beta t} \end{array}\\ \mu_{\beta t}\\ \mu_{ht} \end{array}\right]\sim \left[0,\left[\begin{array}{ccc} I & 0 & \begin{array}{cc} 0 & 0 \end{array}\\ 0 & \Sigma_{\beta } & \begin{array}{cc} 0 & 0 \end{array}\\ \begin{array}{c} 0\\ 0 \end{array} & \begin{array}{c} 0\\ 0 \end{array} & \begin{array}{cc} \begin{array}{c} \Sigma_{a}\\ 0 \end{array} & \begin{array}{c} 0\\ \Sigma_{h} \end{array} \end{array} \end{array}\right]\right]$$

To note, $\Sigma_{\beta }$, $\Sigma_{a}$, and $\Sigma_{h}$ are diagonal matrices, and the parameters $\beta_{t}$, $\beta_{a}$, and $\beta_{h}$ are not correlated with each other.

In this paper, we used R to build a TVP-VAR model. The order of variables entering the model was finally determined as (TEM, RHU, PRE, PAR). The lag-order, *p*, was determined to be 3, 4, 4, 2, 3, and 3 in the ALF, DHF, GGF, HSF, HTF, and SNF regions, respectively.

##### Time-Varying Impulse Response Function

δ(t) represents the change in climate factor per unit, and $x_{\delta \left(t\right)}$ is the change in impact on vegetation brought about by a unit climate factor shock at moment t. In the time-varying impulse response analysis, when t→∞, if $\underset{t\to \infty }{\lim}x_{\delta \left(t\right)}=0$, the established model system has some stability [39]. The lag accumulation also tends to be stable.

In this study, the time-varying impulse response function is used to examine the time-lag effect of climate factors on the EVI from the perspective of the full-time series interval and the time-varying impulse response function analysis of each climate factor on the EVI considering the variability of climate factors on EVI under seasonal changes.

#### 2.3.3. Prediction Model and Accuracy Estimation of the Model

In this paper, we used the monthly EVI data from 2007 to 2017 as the training set and the monthly EVI data from 2018 to 2019 as the prediction set to analyze the dynamic response of vegetation in the context of global climate change. Meanwhile, the root mean square error (RMSE) of the quantitative indicators was used to test and verify the reliability of the TVP-VAR model.

RMSE refers to the sum of squares of the deviations between the predicted and true values of the EVI and the sample size m. The root means the square of the ratio. The smaller its value, the better the fit, and the expression is:

(8) $$RMSE=\sqrt{\frac{1}{m}\overset{m}{\underset{t=1}{\sum }}{\left(EVI_{t}-\hat{EVI_{t}}\right)}^{2}}$$

where $EVI_{t}$ denotes the predicted value of the EVI at time t and $\hat{EVI_{t}}$ denotes the actual value of the EVI at time t.

## 3. Results

### 3.1. The Time-Lag Effect of Climate Factors on EVI

**Figure 3:**
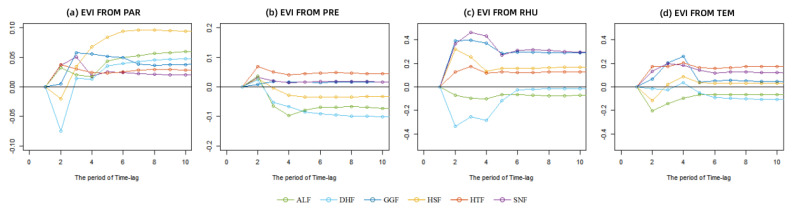
The lag accumulation of PAR (**a**), PRE (**b**), RHU (**c**) and TEM (**d**) on EVI in each eco-station. The y-axis represents the response of EVI to the change of various climate factors.

This section is based on Data set 1 and uses the TVP-VAR model in Section 2.3.2 to study the duration and intensity of climate factors on the EVI time-lag effect and lag accumulation. Due to the spatial heterogeneity of climate conditions, climate factors such as precipitation and temperature have different effects on vegetation growth. The results are shown in Figure 3. Table 3 shows the time-lag effect values and accumulative lag values of each lag period. It shows that the time-lag effect of each climate factor on EVI disappears within six months, and the lag accumulation tends to be stable. However, due to the spatial heterogeneity of climate conditions, climate factors such as precipitation and temperature on vegetation growth are not the same.

TEM showed a time-lag effect of 5.17 ± 0.37 (mean ± standard deviation) months at six field stations and was dominant in effect on vegetation. The time-lag effect of RHU had an impact lasting 4.17 ± 0.69 months. For the SNF ecological stations in the humid north subtropical region, the duration of the time-lag effect of TEM was the longest, with a continuous positive effect of 6 months, and the strongest effect brought by the lag accumulation on EVI (0.13 EVI per unit TEM, in the stable lag-accumulation period); the time-lag effect of RHU was always positive on EVI, but the duration was short, and the time lag disappeared within 4 months. The effect of lag accumulation was stronger than that in the central subtropical region (0.32 EVI per unit RHU). The time-lag effect of TEM on EVI at the ALF and DHF stations in the south subtropical region was a continuous negative response with an average lag time of 4.45 ± 1.09 months, showing a trend of the most decisive influence (median −0.11 EVI per unit TEM, in the first lag-period) to the lowest influence (median −0.015 EVI per unit TEM, in the fifth lag-period). The effect of RHU on EVI was gradually strengthened, showing a trend of the lowest influence (median −0.21 EVI per unit RHU, in the first lag-period) to the strongest influence (median −0.11 EVI per unit RHU, in the fourth lag-period). Differently from the above two ecological stations located in the southern subtropical region, the response of EVI to TEM and RHU changes at the HSF station is positive in the long term. HSF and DHF have similar geographical locations, but the air temperature and humidity bring opposite effects on EVI in the region. From the analysis of the reviewed data, it is known that the vegetation in the HSF region mainly consists of artificial Horsetail pine sparse forest and barren subtropical slope [40], and the vegetation community in the DHF region is subtropical monsoonal evergreen broad-leaved forest [41]. The barren grassland and pine forest are mostly light-loving and drought-tolerant. The ambient humidity is low, which is easier to reduce and tolerate high temperature and humidity compared with the broad-leaved forest environment. That means the accumulation of TEM can promote vegetation growth in areas with lower temperatures. However, high TEM increases the atmospheric demand for evapotranspiration in areas with persistently high temperatures due to vegetation cover type, which inhibits vegetation growth. The accumulation of TEM shows a negative correlation with vegetation growth, which is consistent with the findings of Bei et al. and Lian et al. [42,43].

The mean lag time of the PRE time-lag effect was 5.17 ± 1.07 months. PRE’s lag accumulation was generally weaker in the north and central subtropical region (0.02 EVI/unit PRE and median 0.035 EVI/unit PRE) than in the south subtropical region (−0.063 EVI/unit PRE). However, all study sites in the South Subtropical region showed a negative EVI response to the previous year. Among them, the DHF region showed the strongest negative (−0.09 EVI/unit PRE) and the HSF region showed the lowest negative (−0.03 EVI/unit PRE). The EVI response to PRE is negative because of the high humidity in the regional environment and the large amount of precipitation at the sites in the southern subtropical, which can suppress vegetation growth. Due to its vegetation conditions, the HSF station has weak water-holding capacity and infiltration capacity [44,45]. Even in a humid environment, the HSF station can still maintain soil stability and reduce the influence of precipitation on vegetation growth.

PAR is the energy source for vegetation life activities, affecting vegetation growth and development and regulating vegetation photosynthesis [46]. The average lag time for the time-lag effect of PAR was 5.50 ± 1.11 months. In the first phases of the time lag, PAR had a negative effect on vegetation growth in the southern subtropical ecological stations, DHF (−0.08 EVI/unit PAR) and HSF (−0.02 EVI/unit PAR), since vegetation in these regions is affected by solar radiation for a more extended period during growth and the intensity of solar radiation is higher than other regions. Vegetation under strong PAR will reach light saturation, occur photoinhibition, and manifest as inhibition of photosynthesis and stunted vegetation growth [47]. However, when the hysteresis accumulation brought by PAR reached stability, it all played a role in promoting the growth of vegetation, and the regional differences were slight.

In terms of duration, the time-lag effect of each climate factor on the EVI disappeared within half a year. The difference in the duration of the time-lag effect between field stations in different ecogeographic regions was small (within 1.47 months). From the intensity of the time-lag effect, the responses of the EVI to different climate factors are significantly different. RHU has the greatest impact on the EVI, and this was more obvious in the early stage. In the first lag period, the mean of the absolute value of the lagged effect of each unit of RHU, TEM, PAR, and PRE was 0.28, 0.117, 0.036, and 0.035, respectively. As time goes by, the intensity of the time-lag effect continues to decrease in fluctuation, and the accumulation of lag tends to be stable. From the perspective of spatial distribution, the intensity of time-lag effects of climate factors varies by geographic region. At the SNF station located in the northern subtropical region, the time-lag effects of various climate factors on the EVI all changed from a strong positive effect in the early stage to a gradually weakened negative effect. On the whole, however, the rising and fluctuation of climate factors played a role in promoting the vegetation growth at sites in the northern subtropical region (i.e., the lag-accumulation > 0). Compared with other sites, the vegetation of the SNF site was more sensitive to changes in temperature and humidity (0.32 EVI per unit TEM and 0.13 EVI per unit TEM in the stable lag-accumulation). Similar to the SNF station, changes in climate factors ultimately promoted the growth of vegetation in HTF and GGF stations. The ALF, DHF, and HSF stations are all located in the southern subtropics. Among them, the forest vegetation at the DHF and ALF stations had relatively similar responses to changes in climate factors. The effects of changes in RHU, TEM, and PRE on vegetation were shown as growth inhibition. In contrast to the above two field stations, the responses of the EVI at the HSF station to changes in TEM and RHU were positive in the long run. The HSF and DHF stations are geographically similar, but the effects of air temperature and humidity on the EVI within the region are diametrically opposite.

### 3.2. Sensitivity of Growing Season EVI to Changes in Climate Factor

The vegetation growth season refers to the period in which the biologically effective temperature required for plant growth and development can be guaranteed. In recent years, more and more scholars have started to focus on the relationship between the onset of the vegetation growing season and the changes in climate factors [48,49]. Based on Data set 2, this section uses the time-varying impulse response function to input the TEM, PRE, RHU, and PAR at different stages of the growing season into the model. The response mechanism of forest vegetation growth to climate change in different subtropical regions was studied through the output responses (i.e., the time-delay effects of climate factors in different growing seasons). When the impulse response of the EVI to a climate factor was a positive impulse, it meant that the climate factor promoted vegetation growth; a negative impulse meant that vegetation growth was suppressed. In addition, the magnitude of the positive and negative impulses of the hysteresis effect can reflect the degree of vegetation-growth promotion or suppression, then determine the growth season stage of vegetation, and provide information as to the response of the EVI to climate change under different growth stages.

**Figure 4:**
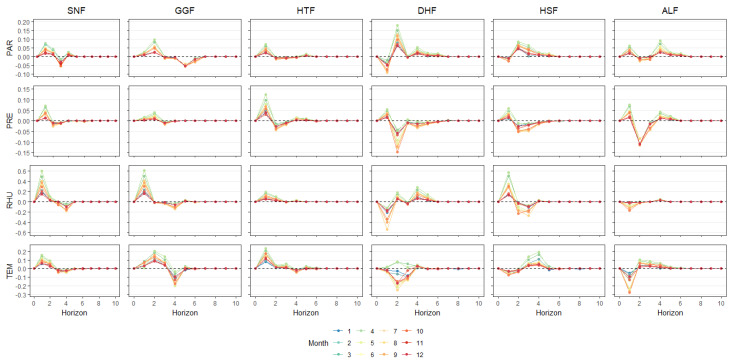
Results of EVI time-varying impulse responses at different time points. The x-axis is the length of impulse response (each period is one month).

The law of lag effect in the northern subtropical and central subtropical regions is more obvious. As shown in Figure 4, the changing trend of impulse response function in different months is close, but there are differences in the intensity of impulse responses. With the same lag phase, the positive impulse brought by each climate factor to vegetation EVI in March and April was the strongest (i.e., the promotion effect of climate factor on vegetation growth is the strongest in this phase). The intensity of the lag effect is more similar from May to October, and the lag effect is weaker from November to February of the following year. More similarly, the hysteresis effect is weaker from November to February. The increase in warmth and precipitation at the start of the growing season caused vegetation to grow swiftly, and vegetation coverage in the north and middle subtropical regions increased dramatically. The lag effect of the EVI in the early growing season was higher than that in the flourishing period, and it had more prolonged effects on plant growth.

The EVI of vegetation in the south subtropical region showed no obvious lag. In addition to climate conditions, vegetation cover types and regional topography also influenced the hysteresis effect in the south subtropical region. The latter’s effects are more pronounced than those of other subtropical regions. At the initial stage of the lag effect, the EVI exhibited a negative impulse response to the mean lag effect of climate factors other than PRE in the south subtropical region. However, DHF exhibited positive impulse responses during the first and the second time-lag effects in March and April. By comparing the HSF station, which is geographically similar to the DHF station, the time-lag effect was not the same as the change at the beginning of the growing season in DHF. Although the two geographical locations are similar, there is a significant difference in altitude. The average altitude of DHF is 750 m, while HSF is 80 m. It can be inferred that the temperature of DHF vegetation is closer to that of the subtropical forest system according to the temperature decrease of 0.8 °C for every 100 m rise in altitude [50]. The increase in temperature and precipitation in the spring promoted vegetation growth. With the increase in altitude, the human disturbance factors decreased, and the vegetation growth environment tended to be better. For the southern subtropical region with high vegetation cover and relatively small EVI changes, the higher EVI accelerates the water transpiration of vegetation at the beginning of the growing season. Transpiration takes away part of the heat, making the EVI in the southern subtropical region negatively correlated with TEM at the beginning of the growing season. Although sufficient precipitation in summer ensures the demand for vegetation growth, high summer temperature also affects air humidity, which becomes a limiting factor for vegetation growth. Thus, the interannual distribution of temperature and precipitation influences the vegetation growth at this site. However, the high temperature and rainfall in summer and autumn inhibit vegetation growth. In winter, when precipitation is low, the increase in temperature is usually accompanied by a decrease in soil water content and air humidity, inhibiting vegetation growth.

At the start of the growing season for forest plants in subtropical areas, plant growth was fast, and the weather had the most obvious effect on plant growth. The EVI was most affected by TEM, followed by RHU. This phenomenon is consistent with the research of Chu et al. [51]. Spring phenology changes are generally more responsive to rising temperatures. Plant growth slowed down in the middle of the growing season, and RHU had a bigger effect on the EVI than TEM. During the non-growing season and at the end of the growing season, the correlation between the EVI of vegetation and climate change was weakest, and each climate factor had a weak lag effect. The correlation between vegetation EVI and climate change was the lowest at the end of the growing season and the non-growing season, and the lagged effect of each factor was weak.

### 3.3. Model Performance for EVI Prediction among Different Field Stations

**Figure 5:**
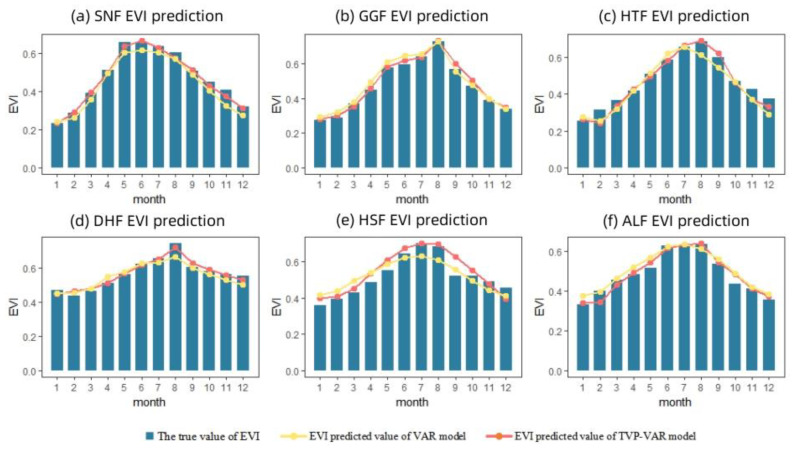
Comparison of prediction results between the VAR model and the TVP-VAR model in SNF (**a**), GGF (**b**), HTF (**c**), DHF (**d**), HSF (**e**) and ALF (**f**).

Compared with VAR models, TVP-VAR models can more effectively capture nonlinear relationships among variables [52]. This paper quantified the time-lag effect, lag accumulation, and time-varying impulse responses of climate factors on the enhanced vegetation index of forest ecological stations in humid subtropical regions of China using the TVP-VAR model. Based on Data set 3, the comparison with the predicted results of the VAR model is added to evaluate the applicability of the TVP-VAR model and explain the necessity of time-varying coefficients in modeling. The prediction accuracy of the TVP-VAR model is shown in Table 4. The comparison result with the VAR model is shown in Figure 5.

Table 4 showed that the TVP-VAR model has high prediction accuracy and small error (RMSE ≤ 0.05). When time-varying parameters are added to a typical VAR model, the model’s prediction accuracy improves by 14.81% on average. The fitting degree of the TVP-VAR model and the real values obtained were substantially greater than for the VAR model, especially in spring and summer when the climate changes (i.e., from March to August; see Figure 5). As an extended VAR model, the TVP-VAR model retains the practical advantages of the VAR model and provides a framework for overcoming the simultaneity problem between variables, and it separates the dynamic impact of each variable on other variables. Due to the addition of time-varying parameters, the relationship characteristics between climate elements and the EVI in various climate backgrounds can be represented with greater precision, hence enhancing the estimation performance of the model.

### 3.4. Comparison of Model Accuracy under the Influence of Extreme Weather

**Figure 6:**
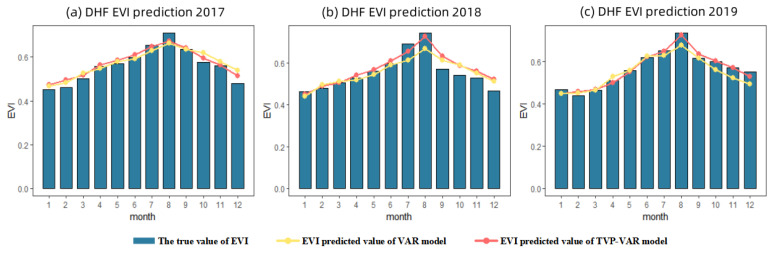
Comparison of 2017 (**a**), 2018 (**b**) and 2019 (**c**) EVI prediction results between VAR model and TVP-VAR model.

The results in Section 3.3 showed that the TVP-VAR model has a better prediction accuracy improvement effect in the DHF station. Our preliminary analysis suggested that this was influenced by the extreme weather of Typhoon Mangkhut in September 2018. Then Data sets 2 and 3 are used to forecast local EVI for 2017–2019 based on DHF station data. The simulation results of TVP-VAR model are shown in Figure 6. The comparison with the prediction results of VAR model is shown in Table 5.

In Table 5, it is showed that in 2019, the TVP-VAR model has the most obvious improvement in the prediction accuracy compared with the traditional model. Compared with 2017 and 2018, after adding time-varying parameters, the accuracy improvement of the model is 58.0% and 145.5% higher, respectively. Without the support of other extreme climate records, we preliminarily believe that the TVP-VAR model has a better performance in the occurrence of extreme climate events.

## 4. Discussion

Prior studies that have noted the effect of climatic factors’ time lag on vegetation growth and development. But these studies are usually conducted on large scales of space and time [2,19]. Very little was found in the literature on the question of investigating the impact of phenological changes and extreme climates on forest growth.

The present study was designed to determine the relationship between EVI and the lag effect of climate factors at different field stations in subtropical regions. The TVP-VAR model was established based on the meteorological monitoring data and EVI data of six typical forest ecological stations in the subtropical region of China from 2007 to 2019. Constrained by the principle of data integrity and considering the nature of the research object, this paper chooses to discuss the time-lag effect of climate factors on vegetation growth only on the monthly scale. Here, we need to make an additional note that the change of time scale does not affect the working of the TVP-VAR model. To prove this point of view, we have added experiments to explore the time-lag effect between air temperature and ground temperature based on daily and monthly scales. Experiments show that the impulse response function can be correctly acted on different time scales. When we determine the time scale of the study, we need to consider the actual situation of the research object. If the scale is too small, frequent changes of variables will affect the stability of the research system, and it is easy to hide important information; if the time scale is too large, it is easy to cause the loss of detailed information, and it is difficult to observe significant changes in the process. As far as the current development of the TVP-VAR model is concerned, there is no professional guidance or explanation for the forestry field. By combining the action process of time-varying impulse response function, we further prove the advantages and applicability of the model in the study of time lag. Impulse response is utilized to analyze and investigate the effect of a shock on a variable during various time intervals. In the initial phase of the model, a shock of one-unit standard deviation will be applied to each climate element, followed by an examination of the EVI’s response to the shock. When the effects of climate change diminish gradually, the response of plant growth to it tends to stabilize. The interpretation of the time-varying pulse results in this paper is based on the analysis of the relevant knowledge of forestry and the mechanism of the impulse response function. 

Based on the long-term monitoring data of six subtropical forest field stations in China, our study shows that the hysteresis effect caused by temperature and humidity conditions is the strongest and lasts relatively longer. The time-lag effect of precipitation is relatively weak. Consistent with our conclusion, Wu et al. [53] analyzed the time-lag effect of climate factors on a global scale and found that the time-lag effect of temperature is the most obvious. The responses of vegetation EVI to climate factors in subtropical regions were significantly different in space. Precipitation is closely related to vegetation greenness. Some scholars in Jiangle County, Fujian Province, found that the forest environment in subtropical regions has high humidity, and when there is more precipitation, the growth of vegetation is easily inhibited. This is consistent with our results. Affected by the regional annual precipitation, the EVI of the field stations located in the southern subtropical region showed a negative response to the PRE. In addition, the responses of vegetation EVI to climate factors in subtropical regions also had significant differences in time. In subtropical regions, the initial period of vegetation growth season is short, mostly in March-April. The lag effect caused by climate factors at the beginning of the growing season was stronger than that at the peak of the growing season. During the growing season, air temperature and humidity are key factors affecting vegetation cover, and temperature changes in the spring play a decisive role in the start of the forest growing season in subtropical regions. In different growing season stages, the difference of the hysteresis effect of climate factors is more obvious in the forest ecological station of north subtropical and central subtropical. The changes of the impulse responses at different growth season stages in the field station in the south subtropical region were small, with only a slight difference in numerical value. Therefore, the change of the growth season of forest trees in the south subtropical region was more stable than that in the northern subtropical and southern subtropical regions. For areas with ecosystem degradation such as HSF, when forest managers carry out large-scale vegetation restoration, they can consider the natural conditions that restrict the growth and development of forest plant communities according to climate factors and combine the local environmental carrying capacity to synchronize the growth season and growth of vegetation. Adequate precipitation, improved water use efficiency, and selection of a suitable environment for vegetation growth can all achieve the diversification and reconstruction of forest ecosystems [47].

Compared with the traditional VAR model, the prediction accuracy of TVP-VAR in DHF field station in Table 3 had a more obvious improvement. In response to this finding, we set up supplementary experiments in Section 3.4. The TVP-VAR model has a higher prediction accuracy improvement compared with the VAR model when forecasting the 2019 EVI numerical value compared with the years before the typhoon. This shows that compared with the traditional model, the ability of the model to cope with sudden shocks is enhanced after adding time-varying parameters. The TVP-VAR model is more sensitive to capture short-term ecosystem changes. The finding could help predict forest growth trends under extreme climatic conditions. In the future, supported by new data, we will add other mutation-producing time points to the time-varying impulse response part to further demonstrate the high applicability of the TVP-VAR model in community succession forest systems. At the same time, it can also provide reference for the application of TVP-VAR model in forestry and meteorological fields. TVP-VAR is an econometric model. Therefore, there are also limitations inherent in general econometric models. That is, it is difficult to deal with high-dimensional, complex and unstructured data. When faced with observation data sets with increased dimensions and increased data volume, based on the TVP-VAR model, clustering, dimensionality reduction, and other methods can be used to optimize and improve the work efficiency of the model, or combine econometric models with neural network models. In this way, the complexity of the model can be reduced on the basis of ensuring the robustness of the model and further improve the reliability of the results and the interpretability of the model.

## 5. Conclusions

In subtropical regions, air humidity and temperature are the factors that have the greatest impact on vegetation growth. We found that the responses of vegetation EVI to climate factors varied significantly in time and space even though all ecological stations were located in subtropical regions. In addition, TEM and RHU are key factors affecting vegetation growth. As far as forest vegetation phenology is concerned, TEM plays a decisive role in the changes of vegetation growth season stages. Verified by the data set, the TVP-VAR model has high accuracy. The impact of emergencies can be better captured. This shows that the TVP-VAR model can not only analyze the hysteresis effect brought by climatic factors but also predict the vegetation growth status in subtropical regions of China in combination with climate change analysis. In this case, it will provide technical support with high applicability and reliable data for sustainable forest management and estimation of potential carbon sinks. In addition, in the context of global climate change, if the vegetation coverage is to be increased and the ecological environment is to be further improved, specific human intervention measures need to be incorporated. For example, storage devices could be installed at suitable locations within forest areas to collect water. The use of rainwater accumulation technology to form forest microclimates can change the spatial and temporal distribution of water in the forest area and ensure water for forest use during the dry season in subtropical areas. The results can provide support for im-proving the extensive forest management pattern.

## Figures and Tables

**Table 1:** Basic information for the national field scientific observation and research station for the forest ecosystem.

Ecoregion Type	Eco Station	Abbreviation	Longitude and Latitude	Type of Landform	Average Elevation (m)	Annual Rainfall (mm)
Northern subtropical humid region	Shennongjia	SNF	110°36′ E, 31°68′ N	Mountain	1700	1300~1722
Central subtropical humid region	Minya Konka	GGF	101°59′ E, 29°34′ N	Valley Glacier	3000	1750~2175
	Huitong	HTF	109°30′ E, 26°48′ N	Hilly	700	1200~1400
Southern subtropical humid region	Ailao Mountain	ALF	101°01′ E, 24°32′ N	Mountain	2450	1931
	Dinghu Mountain	DHF	112°31′ E, 23°09′ N	Hilly	600	1564
	Heshan	HSF	112°54′ E, 22°41′ N	Hilly	80	1700

**Table 2:** Long-term meteorological monitoring data for forest ecosystem field stations. The numerical range of air temperature, air humidity, and photosynthetically active radiation is the numerical range of the monthly mean value; the numerical range of the precipitation is the numerical range of the monthly total value.

Eco Station Code	Time Span	Precipitation Range (mm)	Air Temperature Range (°C)	Air Humidity Range (%)	Photosynthetically Active Radiation Range (mol/m^2^)
SNF	2009/01~2019/12	2.3~536.6	−9.6~28.0	71.3~91.8	185.1~1431.3
GGF	2007/01~2019/12	5.2~490.1	−7.9~19.8	81.4~97.0	214.1~898.1
HTF	2007/01~2019/12	0.0~418.7	−1.8~33.3	69.8~95.3	138.6~1260.2
ALF	2007/01~2019/12	0.0~540.9	3.8~16.6	63.3~96.5	354.0~1259.7
DHF	2007/01~2019/12	0.0~547.8	9.2~35.3	62.9~90.0	261.1~1186.9
HSF	2007/01~2019/12	0.0~563.5	9.6~36.6	56.7~98.1	87.1~1290.9

**Table 3:** The time−lag effects of different climate factors on the EVI of each forest ecological field station under different lag periods.

	Station	The Lag-Period						The Lag-Accumulation
Value		1	2	3	4	5	6	
PAR	SNF	0.04	0.01	−0.03	0.01	0.00	0.00	0.02
	GGF	−0.01	0.05	0.00	0.00	0.00	−0.01	0.04
	HTF	0.04	−0.01	0.00	0.00	0.00	0.00	0.03
	ALF	0.03	−0.01	0.00	0.03	0.00	0.01	0.05
	DHF	−0.08	0.09	0.00	0.02	0.01	0.00	0.04
	HSF	−0.02	0.05	0.03	0.01	0.01	0.00	0.09
PRE	SNF	0.03	−0.01	−0.01	0.00	0.00	0.00	0.02
	GGF	0.01	0.01	0.00	0.00	0.00	0.00	0.02
	HTF	0.07	−0.02	−0.01	0.00	0.00	0.00	0.05
	ALF	0.04	−0.10	−0.04	0.02	0.01	0.00	−0.07
	DHF	0.03	−0.08	−0.01	−0.02	−0.01	0.00	−0.09
	HSF	0.03	−0.03	−0.03	0.00	0.00	0.00	−0.03
RHU	SNF	0.36	0.11	−0.04	−0.16	0.04	0.01	0.32
	GGF	0.39	0.00	−0.03	−0.08	0.01	0.00	0.29
	HTF	0.13	0.04	−0.06	0.01	0.00	0.00	0.12
	ALF	−0.07	−0.03	0.00	0.04	0.00	0.00	−0.07
	DHF	−0.34	0.08	−0.03	0.17	0.09	0.01	−0.02
	HSF	0.39	−0.06	−0.12	0.02	0.00	0.00	0.16
TEM	SNF	0.13	0.07	−0.02	−0.04	−0.02	0.01	0.13
	GGF	0.06	0.13	0.06	−0.21	0.01	0.00	0.05
	HTF	0.17	0.00	0.03	−0.03	0.00	0.00	0.16
	ALF	−0.20	0.05	0.05	0.02	0.01	0.00	−0.07
	DHF	−0.02	−0.01	0.07	−0.09	−0.04	0.00	−0.10
	HSF	−0.12	0.14	0.07	−0.05	0.00	0.00	0.03

**Table 4:** Comparison of prediction accuracy between the VAR model and the TVP-VAR model.

Eco Station	RMSE of the Predicted Value		Improvement of Prediction Accuracy (%)
	VAR	TVP-VAR	
SNF	0.05631	0.04556	19.09
GGF	0.05219	0.04635	11.19
HTF	0.05366	0.04813	10.31
ALF	0.05903	0.05145	12.84
DHF	0.06516	0.04926	24.40
HSF	0.05055	0.04486	11.06
Average	0.05615	0.04760	14.81

**Table 5:** Comparison of prediction accuracy between VAR model and TVP-VAR model.

Year	RMSE of the Predicted Value		Improvement of Prediction Accuracy (%)
	VAR	TVP-VAR	
2017	0.05952	0.05033	15.44
2018	0.08223	0.07405	9.94
2019	0.06516	0.04926	24.40
Average	0.06900	0.05788	16.11

## Data Availability

The data that support the findings of this study are openly available in “National Ecosystem Observation and Research Network of China” (http://www.cnern.org/index.action, accessed on 12 April 2021) and “NASA” (https://earthdata.nasa.gov/, accessed on 12 April 2021).
